# Elucidation of physicochemical properties of polysaccharides extracted from *Cordyceps militaris* fruiting bodies with different drying treatments and their effects on ulcerative colitis in zebrafish

**DOI:** 10.3389/fnut.2022.980357

**Published:** 2022-09-02

**Authors:** Yin Wei, Xiao Du, Yangbian Guo, Mingchang Chang, Bing Deng, Jingyu Liu, Jinling Cao

**Affiliations:** ^1^College of Food Science and Engineering, Shanxi Agricultural University, Jinzhong, China; ^2^Shanxi Key Laboratory of Edible Fungi for Loess Plateau, Taiyuan, China; ^3^Collaborative Innovation Center of Quality and Efficiency of Loess Plateau Edible Fungi, Jinzhong, China

**Keywords:** *Cordyceps militaris*, polysaccharides, drying method, ulcerative colitis, zebrafish

## Abstract

Dry fruiting bodies of *Cordyceps militaris* (CMF) have been widely used in folk tonic foods and traditional herbal medicine in East Asia. Drying treatment serves as the last step in CMF industrial processes. In this work, the physicochemical properties of polysaccharides from *C. militaris* fruiting bodies (CMFPs) with hot air drying (HD), far-infrared radiation drying (ID) and vacuum freeze-drying (FD) treatments were analyzed, and their effects on ulcerative colitis (UC) were further investigated in oxazolone-induced zebrafish. The results showed that physicochemical properties of CMFP-H, CMFP-I and CMFP-F were obvious different. CMFPs could repair the intestinal mucosal barrier, inhibit ROS generation and the activities of MDA and MPO, and improve the activities of SOD, CAT, ACP, AKP and LZM. Further detection indicated that CMFPs could better improve UC *via* activating the MyD88/NF-κB signaling pathway *in vivo*. However, CMFP-H, CMFP-F and CMFP-I exhibited diverse regulation effects on specific immune-related enzymes and cytokines. The data would be helpful for finding practical and rapid drying methods for macro-fungi and further exploring CMFPs as functional food ingredients or complementary medicines for the treatments of UC.

## Introduction

*Cordyceps militaris* (*C. militaris*) is a rare medicinal entomopathogenic fungus. At present, *C. militaris*, one of the widely-range industrialized cultivation mushrooms, has been recognized as a potentially excellent raw material for functional foods or alternative drugs with immune regulation, anti-oxidation, anti-tumor, and blood sugar regulation (1–3). Its dry fruiting bodies have been widely used in folk tonic foods and traditional herbal medicine in East Asia (4). The dry fruiting bodies of *C. militaris* were approved as a new resource food material by the National Health Commission (NHC) of the People's Republic of China in early 2009, and now are widely used in direct consumption, wine, canned food, condiments, and beverages in China (5). It is well-known that drying treatment has been widely used to prolong the storage time and shelf life extension of food production (6). Hot air drying (HD), far-infrared radiation drying (ID), and vacuum freeze-drying (FD) are the three most popular drying methods, which are mainly used in the pretreatment of raw food materials (7). However, growing studies have indicated that the rapid drying process had a significant influence on the physical and chemical properties of food materials (8, 9). At present, drying treatment, serves as the last step in *C. militaris* industrial processes, plays a key role in controlling the quality of its dry fruiting body production. However, the underlying effects of different drying treatments on physicochemical characterization of *C. militaris* fruiting bodies, especially natural bioactive components, is lacking until now.

Inflammatory bowel disease (IBD) is a common chronic intestinal inflammatory disease characterized by chronic and uncontrolled intestinal mucosa inflammation, affecting any part of the intestines and stomach (10). The two most common types of IBD are Crohn's disease (CD) and ulcerative colitis (UC) (11). They are both characterized by an abnormal chronic or recurring immune response, which leads to tissue injury with bowel inflammation and ulceration (12). The cytotoxic agents, such as steroids, immunomodulators, and surgery, are widely used to treat IBD. However, it is necessary to explore other therapeutic options due to the high cost and side effects of these agents, and their non-specific efficacy (13). The data of existing studies have indicated that mushroom polysaccharides are gradually considered a potentially powerful immune-modulator source for the treatments of IBD (14, 15). Several studies have shown that the polysaccharides extracted from dried *C. militaris* fruiting bodies (CMFPs) have been discovered and explored in numerous aspects, such as immune-regulation, anti-inflammation, macrophage activation, and anti-tumor (1–3). Accumulating evidence suggested zebrafish could serve as an ideal animal model to study IBD and gastrointestinal diseases due to their high similarity of genes and gastrointestinal system to humans (16, 17). Therefore, the effects of CMFPs from different drying treatments on zebrafish colitis were investigated *in vivo* in the present study. The data might be helpful to a thorough understand of the alleviating mechanism of fruit body polysaccharides processed by different drying methods on colitis.

Here, this work was designed to systematically assess the physicochemical characterization differences of CMFPs from HD, ID, and FD treatments and their beneficial effects on colitis in zebrafish *in vivo*. This will provide valuable information for finding practical and rapid drying methods for this macro-fungi and further exploring CMFPs as functional food ingredients or complementary medicines for the treatments of IBD.

## Materials and methods

### Materials and chemical reagents

Fresh fruiting bodies of *C. militaris* (Shanxi Key Laboratory of Edible Fungi on Loess Plateau, China) were identified as Cordycipitaceae fungus *Cordyceps militaris* by Professor Jingyu Liu of Shanxi Agricultural University. Folin, gallic acid, DEAE-cellulose-52, and AB-PAS staining kits were purchased from Solarbio (Beijing, China). Galacturonic acid was purchased from Sigma-Aldrich (USA). Chromatographic grade trifluoroacetic acid (TFA) was purchased from Aladdin (Shanghai, China). Sephacryl S-400 was purchased from GE Healthcare (USA). Four-ethoxymethylene-2-phenylazolin-5-one (oxazolone) was purchased from Alfa Aesar Chemical Co., Ltd. (Tianjin, China). Five-aminosalicylic acid (mesalazine) was purchased from Macleans Biochemical Technology Co., Ltd. (Shanghai, China). Protein quantitative detection kits, reactive oxygen species (ROS), superoxide dismutase (SOD), catalase (CAT), malondialdehyde (MDA), acid phosphatase (ACP), alkaline phosphatase (AKP), lysozyme (LZM) and myeloperoxidase (MPO) test kits were purchased from Nanjing Jiancheng Institute of Bioengineering (Nanjing, China). All other reagents are of analytical grade.

### Drying treatments of *C. militaris* fruiting-bodies

Fresh and relatively uniform size *C. militaris* fruiting bodies (CMFs) without diseases, pests, and mildew were selected and immediately dried using three drying methods (HD, FD, and ID). HD treatment was conducted in an electric thermostatic air-blast drying oven (GZX-9240MBE, Bosun Industry Co., Ltd., China) at 50°C. ID treatment was carried out in a far-infrared rapid constant temperature drying oven (HD-E804-45A, Shanghai Yuejin Medical Machinery Co., Ltd., China) at 50°C. The FD group, fresh CMFs were pre-cooled at 4°C for 2 h, quick-frozen at−20°C for 12 h, and finally frozen at −80°C and below 1 kPa in vacuum freeze-drying treatment (Alpha2-4LSCbasic, CHRIST, Germany). During the dying, samples were taken at intervals of 4 h to determine the moisture content of CMFs. When the moisture content dropped to approximately 5%, the drying was terminated. Next, the homogeneous powders of CMFs were obtained by grinding through an 80-mesh sieve and stored at 4°C, respectively.

### Extraction of CMFPs

The extraction of CMFPs wes performed according to our previously published protocols (18). Briefly, 100 g powders of different dried CMFs and 4 L ultrapure water (Milli-Q ^®^ Reference, Merck, Germany) were mixed and incubated at 80°C for 3 h. After centrifugation (5804 R, Eppendorf, Germany) at 4,500 *g* for 15 min, the supernatant was collected and concentrated under reduced pressure using an RV-10 rotary evaporator (IKA, Germany). Then, 10.6% potassium ferrocyanide, 21.9% zinc acetate solution, and concentrated filtrate were mixed in a ratio of 1:1:10 to remove protein from the extract. After complete mixing, let stand for 30 min and centrifuge the mixture at 12,000 *g* for 10 min. The supernatant was subsequently concentrated and dialyzed (molecular weight (MW) cutoff of 3.5 KD) with ultrapure water until the conductivity was below 20. Next, a 4-fold volume of ethanol solution was added to the supernatant and incubated at 4°C for 12 h. Finally, the precipitate was collected and lyophilized as CMFPs. CMFPs from different dried CMFs were, respectively named CMFP-H, CMFP-I, and CMFP-F for subsequent studies.

### Physicochemical properties of CMFPs

#### Determination of total carbohydrate, protein, uronic acid, and phenol contents

The yields and purities of CMFP-H, CMFP-I, and CMFP-F were determined by the phenol-sulfuric acid method with different glucose concentrations as the standard, respectively. Subsequently, the contents of total protein, uronic acid, and phenol were determined by the bicinchoninic acid (BCA) method, the carbazole-sulfuric acid method, Folin–Ciocalteu assay (19).

#### Analysis of monosaccharide composition

Five mg CMFPs samples were hydrolyzed with 10 mL of 2 mol/L TFA at 120°C for 3 h in an ampoule then dried under a nitrogen blower (MD200-1, Aosheng Instruments Co., Ltd, China). The monosaccharide composition of CMFP-H, CMFP-I, and CMFP-F was determined using ion chromatography (ICS5000, Thermo Fisher, USA) equipped with carbopac chromatographic column (TMPA20, 3 × 150, Dionex, USA) according to our previous method (18). The column was sequentially eluted with ultrapure water, 250 mmol/L NaOH and a mixture solution (50 mmol/L NaOH and 500 mmol/L NaOAC) at a 0.3 mL/min. The column temperature and injection volume were set at 30°C and 5 μL, respectively.

#### Determination of molecular weight and its distribution

The molecular weight and its the distribution of CMFP-H, CMFP-I, and CMFP-F were measured by high-performance exclusion chromatography (HP-SEC). Briefly, the polysaccharide samples were dissolved in ultrapure water to a concentration of 5 mg/mL, filtered through a 0.22 μm microporous membrane, and then injected into a BRT105-104-102 tandem gel column (8 × 300 mm, China). The column was eluted with 0.05 mol/L NaCl solution at a flow rate of 0.6 mL/min. The column temperature and injection volume were set at 40°C and 20 μL, respectively. The values of dn/dc were detected using a RID-10A differential refraction detector (LC-10A, Shimadzu, Japan).

#### Surface morphology

The morphological characteristics of CMFP-H, CMFP-I, and CMFP-F were observed and compared by a scanning electron microscope (JSM-6490LV, JEOL, Japan).

#### Analysis of the elution characteristics of CMFPs components

The elution curves of CMFP-H, CMFP-I, and CMFP-F components were investigated based on our previous method (18). Briefly, the CMFP-H, CMFP-I, and CMFP-F were loaded on a DEAE-cellulose-52 ion exchange column (2.8 × 33 cm) and then eluted with ultrapure water and 0.1 mol/L NaCl solution at the flow rate of 3 mL/min. The eluted components were sequentially collected according to the absorbance value at 490 nm, were filtered through 0.22 μm microporous membranes, and were reloaded into a Sephacryl-S400 gel column (XK 26/100) of AKTATM pure chromatography system (GE, USA). The column was eluted with 0.2 mol/L NH4HCO3 solutions at a flow rate of 1.3 mL/min. The eluents with the same elution curve peak were combined using an automatic collector, dialyzed, and then freeze-dried to obtain the corresponding components.

### Animal experiments

#### Animals handling

Healthy male 4-month-old zebrafish (*Danio rerio*, AB strain), with an average body length of 3–3.5 cm, were purchased from Shanghai Jiayu Aquarium and were kept in a circulatory system (water temperature, 28 ± 1°C; pH, 7.0–7.2; dissolved oxygen, 6.5 mg/L; photoperiod, light/dark = 14/10 h) to adapt to the laboratory conditions. Fish were fed compound feed for adult zebrafish (Special fish food for small fish, Shandong Weifang Yipin Pet Products Co., Ltd.) (Shandong, China) 3 times a day. All animal work was approved by the Experimental Animal Ethics Committee at Shanxi Agricultural University and carried out by the guidelines for the care and use of experimental animals.

Zebrafish intestinal inflammation model was constructed in accordance with the method of Brugman & Nieuwenhuis (20). Adult zebrafish intestinal inflammation was induced by intrarectal injection of 0.2 % oxazolone (6 μL/g body weight). After oxazolone injection for 5 h, the intestine samples were collected to determine whether the enteritis model was successfully established. On this basis, 270 zebrafish were randomly divided into six groups, including normal control (NC) group (fed untreated feed), model control (MC) group (injected 0.2% oxazolone), positive control (PC) group (injected 0.2% oxazolone + feed containing 6% mesalazine), and three intervention (CMFP-H, CMFP-I, CMFP-F) groups (injected 0.2% oxazolone + feed containing 6% CMFP-H, CMFP-I, CMFP-F). Each group had 45 fish, with 3 repetitions. After treatment for 3 and 6 d, the intestinal tissues in each group were collected immediately for further analysis.

#### Histopathological examination

The intestinal tissue samples were fixed with 10% formalin for 24 h and then rinsed with flowing water for 24 h. Subsequently, after dehydration with a gradient series of ethanol, dimethyl benzene treatment, and paraffin embedding, the embedded blocks were sliced with an automatic rotary slicer (RM 2255, Leica, German) and collected on the glass slide. Afterward, the drops were dyed with hematoxylin and eosin (H.E.) or AB-PAS and mounted with neutral gum. Finally, the intestinal histopathological changes were observed using the optical microscope (Eclipse Ni-U, Nikon, Japan).

#### Biochemical assay

The intestinal tissues were collected and weighed immediately, homogenized in ice-cold saline (1:10, w/v), and centrifuged at 3,500 r/min at 4°C for 10 min. The supernatant obtained was used for to determine of ROS, SOD, CAT, MDA and ACP, AKP, LZM, and MPO according to the manufacturer's instructions with commercial kits (China Nanjing Jiancheng Biotechnology Co., Ltd.) using ultra-micro UV spectrophotometer (NanoDrop One, Thermo, USA). The BCA method was used to detect the protein content of each sample, which was used as the standard to calibrate the level of determined indexes. The 2,7-Dichlorofluorescin Diacetate (DCFH-DA) probe measured the ROS level with the excitation wavelength of 500 nm and the emission wavelength of 525 nm.

#### Real-time fluorescence quantitative PCR analysis

Total RNA was extracted from zebrafish intestinal tissues using RNAiso Plus (TAKARA, Japan) and reverse transcripted into cDNA using PrimeScript™ RT Reagent Kit with gDNA Eraser Kit (TAKARA, Japan). The qRT-PCR reaction was then carried out on a CFC Connect PCR tester (Bio-RAD, USA) to determine the relative expression levels of interleukin-1β (*IL-1*β), tumor necrosis factor-α (*TNF-*α), interferon (*IFN*), interleukin-10 (*IL-10*), *NF-*κ*B P65*, tumor necrosis factor receptor-associated molecule (*TRAF6*) and myeloid differentiation factor 88 (*MyD88*) based on β-actin as a reference gene. The relative mRNA expression levels of each gene were calculated according to the 2^−Δ*ΔCt*^ comparative quantitative method (21). The primers used in this study were designed and synthesized by Okdingsheng Biotechnology Co., Ltd. (Beijing, China) (Supplementary Table 1).

### Statistical analysis

All experiments were repeated three times, and the data were expressed as the mean ±standard deviation (SD). One-way (ANOVA) analysis was used to compare the variation between groups in SPSS software. Graphs were performed using Graph Pad Prism software version 8.0.

The differences were statistically significant when *p* < 0.01 (^**^) and *p* < 0.05 (^*^).

## Results

### Physicochemical properties of CMFPs

As shown in Table 1, CMFP-I had the highest extraction rate (3.61%), followed by CMFP-F (2.60%) and CMFP-H (2.19%). The contents of neutral sugar (85.58%), uronic acid (26.55%), and total phenolic (3.80%) in CMFP-F were higher than those in CMFP-H (73.23, 21.73, 0.57%) and CMFP-I (67.92, 20.55, 1.19%). However, the protein content of CMFP-F (2.84%) was significantly lower than that of CMFP-H (5.01%) and CMFP-I (4.64%). Meanwhile, UV-visible spectral analysis showed that three CMFPs all had weak absorption peaks at 280 nm, but the peak heights differed slightly (data not shown).

**Table 1:** Extraction yields, chemical properties, and constituent monosaccharides of the polysaccharides from fruiting bodies of *Cordyceps militaris* under different drying treatments.

**Sample**	**CMFP-I**	**CMFP-H**	**CMFP-F**
Yield (%)	3.61	2.19	2.60
Carbohydrate (wt%)	67.92	73.23	85.58
Protein (wt%)	4.64	5.01	2.84
Uronic acid (wt%)	20.55	21.73	26.55
Polyphenol (wt%)	1.19	0.57	3.80
**Monosaccharides composition (molar ratios) (mol%)**			
Glucose	5.63	7.13	7.57
Mannose	2.84	1.83	1.6
Galactose	1.48	1	0.81
Glucosamine hydrochloride	0.04	0.04	0.04

The monosaccharide composition of CMFPs from different dried CMFs were further investigated by using IC technique (Supplementary Figure 1). The data showed that CMFP-H, CMFP-I, and CMFP-F were all composed of glucose, mannose, galactose, and glucosamine hydrochloride. However, the molar ratios of the three samples were significantly different. The glucose content of CMFP-F (7.57%) was higher than that of CMFP-I and CMFP-H (5.63% and 7.13%), while its mannose and galactose content was the lowest (Table 1).

### Molecular weight and apparent structure of CMFPs

**Figure 1:**
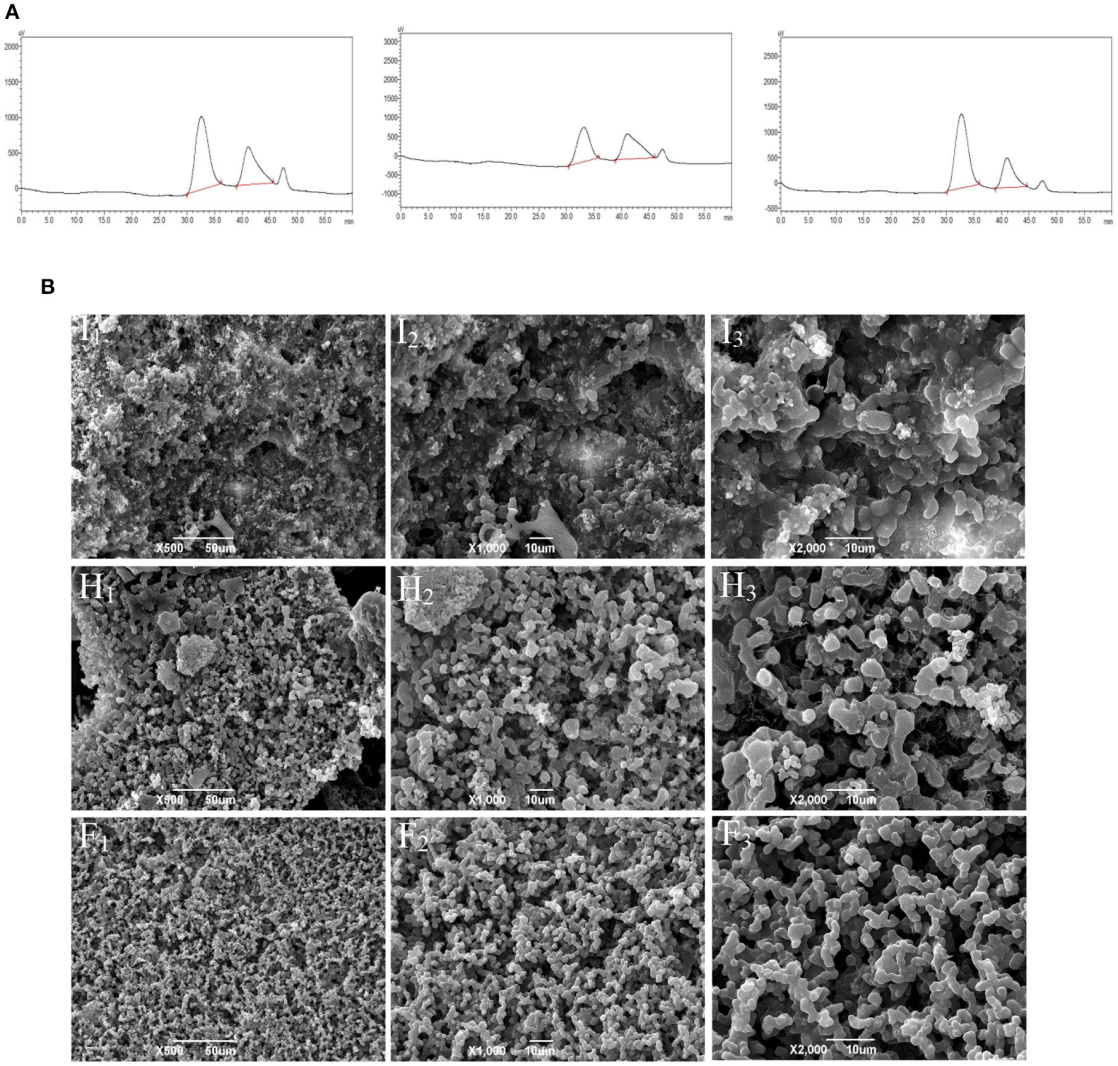
Molecular weight profile and surface morphology of the polysaccharides from fruiting bodies of *Cordyceps militaris* under different drying treatments. **(A)** Molecular weight profiles of CMFPs. **(B)** SEM photomicrographs of CMFPs. (H) CMFP-H. (I) CMFP-I. (F) CMFP-F.

According to the molecular weight spectrum (Figure 1A) determined by HPGPC, CMFP-H, CMFP-I, and CMFP-F had a similar scope and contained two polysaccharide components with similar peak shapes and peak times. The detailed molecular weight size and distribution of CMFP-H, CMFP-I, and CMFP-F were summarized in Supplementary Table 2. The average MW of peak 1 and peak 2 in CMFPs ranged from 10.99848 × 10^5^ Da to 14.19080 × 10^5^ Da and from 2.6533 × 10^4^ Da to 2.8101 × 10^4^ Da. The MW of CMFP-H peak 1 (14.19080 × 10^5^ Da) was significantly higher than the MW of CMFP-F (13.31181 × 10^5^ Da) and CMFP-I (10.99848 × 10^5^ Da) peak 1, while the proportion of CMFP-F (73.166%) was higher than that of CMFP-H (63.796%) and CMFP-I (50.118%). At the same time, the MW of CMFP-F (2.8101 × 10^4^ Da) and CMFP-H (2.7530 × 10^4^ Da) peak 2 was higher than that of CMFP-I (2.6533 × 10^4^ Da), but the proportion of CMFP-F (26.834%) was significantly lower than that of CMFP-H (36.204%) and CMFP-I (49.882%). In addition, the polydispersity indexes of peak 1 and peak 2 of CMFPs were about 1.9 and 1.3.

The results of CMFPs ultrastructure by SEM found significant differences in the structure and morphology of CMFP-H, CMFP-I, and CMFP-F (Figure 1B). The CMFP-I had a relatively rough topographic surface, irregular pore structure, and protrusions, while the CMFP-H had uneven surface particle size with a small amount of debris. Compared with CMFP-H and CMFP-I, CMFP-F had a clear design and a more uniform surface particle size and distribution.

### Separation of CMFPs components

**Figure 2:**
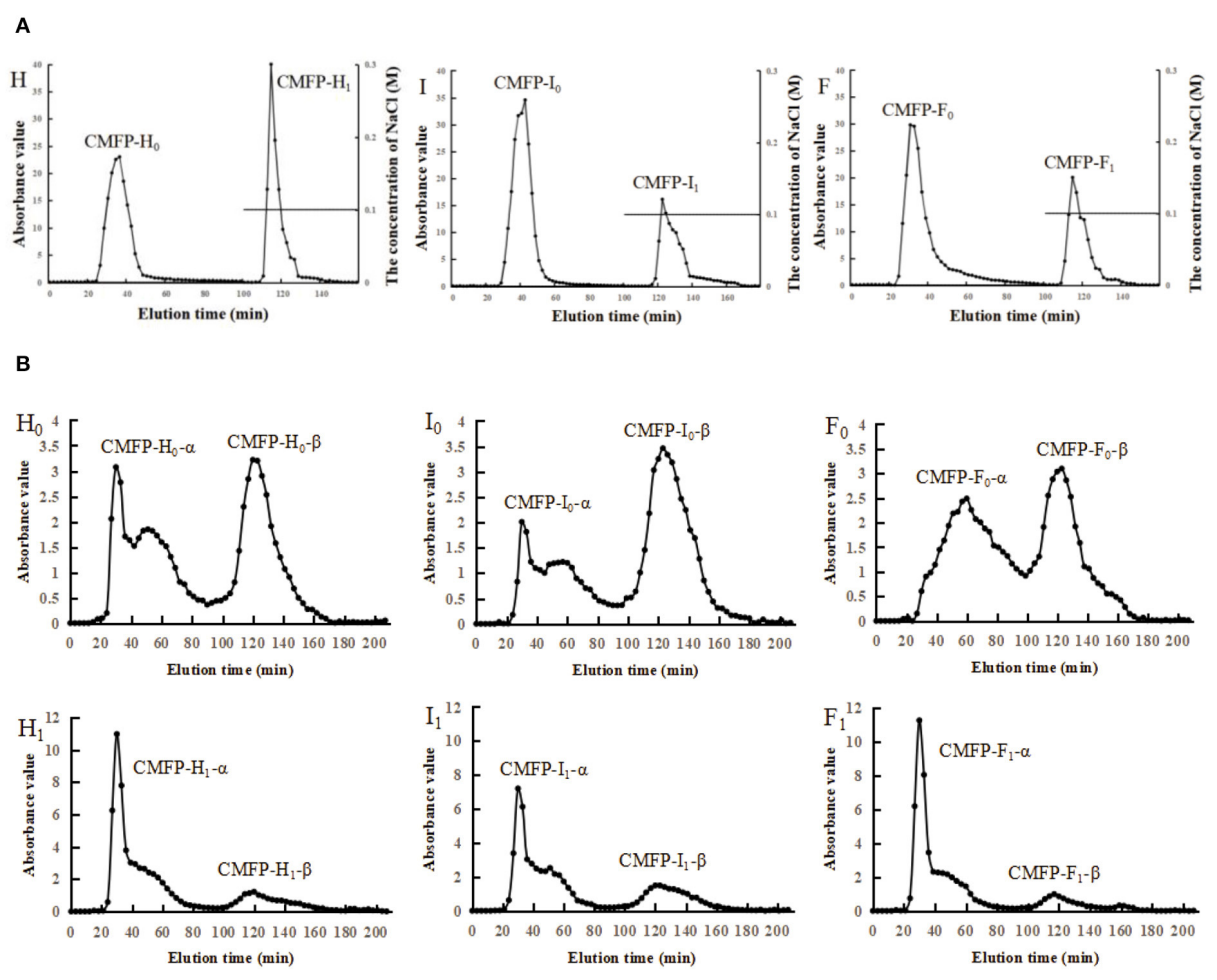
Elution curve of DEAE-52 and Sephacryl S-400 for CMFPs. **(A)** Elution curve of DEAE-52 of CMFPs. **(B)** Elution curve of Sephacryl S-400 of CMFPs. (H) CMFP-H. (I) CMFP-I. (F) CMFP-F. (H_0_) CMFP-H_0_. (I_0_) CMFP-I_0_. (F_0_) CMFP-F_0_. (H_1_) CMFP-H_1_. (I_1_) CMFP-I_1_. (F_1_) CMFP-F_1_.

The elution curve of DEAE-52 is shown in Figure 2. The peak times of the three CMFPs were slightly different, but the peak positions were relatively consistent. After the CMFPs were eluted with the DEAE-52 anion exchange column, two components were obtained, respectively: 0M components (CMFP-H_0_, CMFP-I_0_, and CMFP-F_0_) eluted with distilled water and 0.1M fractions (CMFP-H_1_, CMFP-I_1_, and CMFP-F_1_) eluted with 0.1M NaCl (Figure 2A). Combining the analysis in Supplementary Table 3, the result suggested that the recovery rate of 0M components was as follows: CMFP-H_0_ > CMFP-I_0_ > CMFP-F_0_, while the order of 0.1M components was precisely the opposite, CMFP-F_1_ > CMFP-I_1_ > CMFP- H_1_.

Next, the 0M and 0.1M fractions of polysaccharides were further separated and purified using Sephacryl S-400 gel columns. Each sample eluted two components, namely the α component and the β component (Figure 2B). The two peaks of CMFP-F_0_ were relatively complete and symmetrical, while the peak shapes of the elution peaks of CMFP-H_0_-α and CMFP-I_0_-α were incomplete and not a single symmetrical peak. The time and shape of the peaks in CMFP-H_1_, CMFP-I_1_, and CMFP-F_1_ were similar, but the peak areas were significantly different. Furthermore, the recoveries of each component were analyzed and compared (Supplementary Table 4) to obtain the relative size of the peak area for each component. The relative size of the peak area for 0M components was as follows: CMFP-H_0_-α > CMFP-F_0_-α > CMFP-I_0_-α; CMFP-I_0_-β > CMFP-F_0_-β > CMFP-H_0_-β. While the relative size of the peak area for 0.1M components was: CMFP-F_1_-α > CMFP-I_1_-α > CMFP-H_1_-α; CMFP-H_1_-β > CMFP-I_1_-β > CMFP-F_1_-β.

### Histopathological observation of intestine in colitis zebrafish

**Figure 3:**
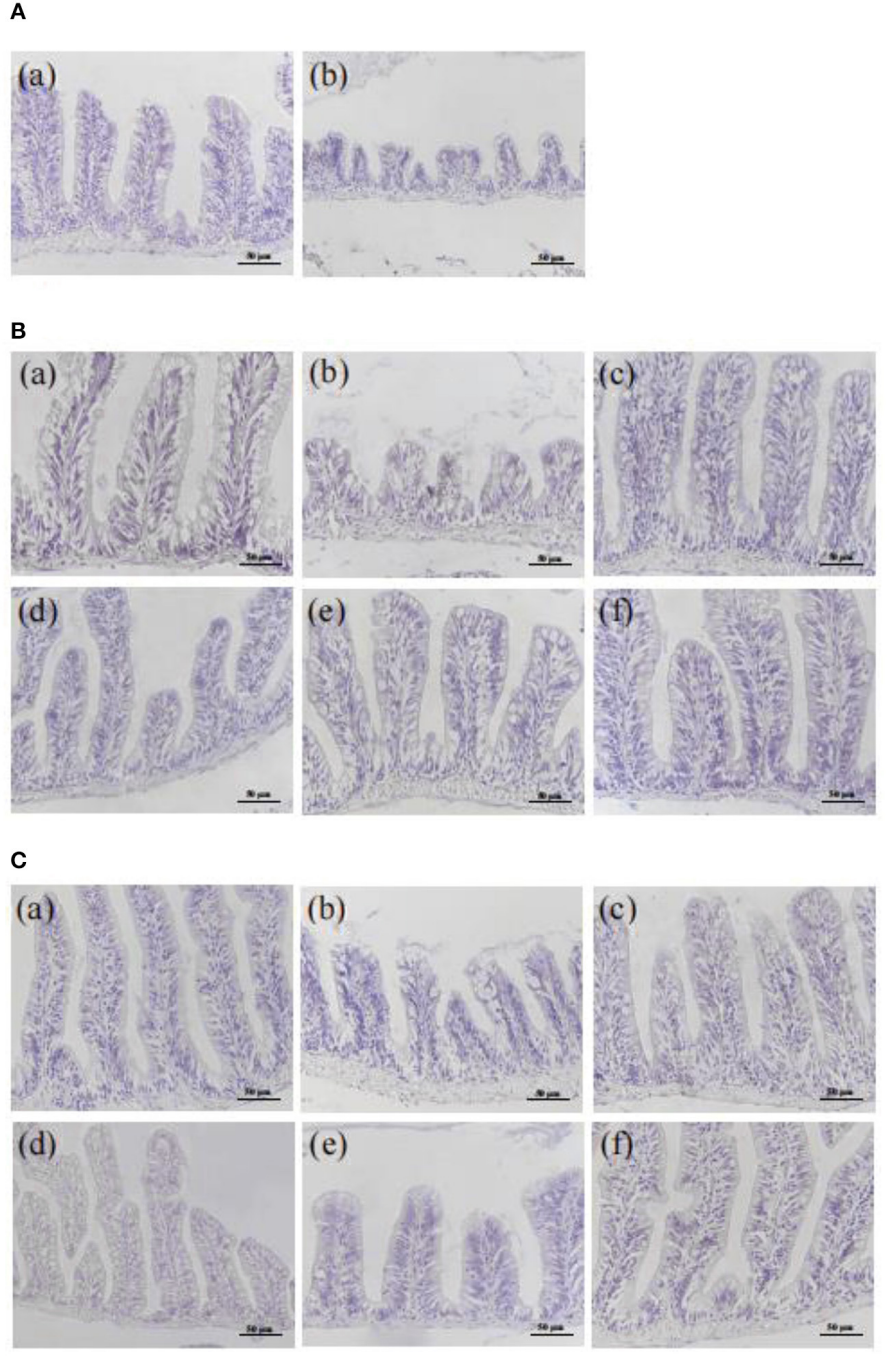
Effect of CMFPs on the histopathological structure (HE staining) of intestinal in inflammatory bowel disease zebrafish. **(A)** 5 h. **(B)** 3 d. **(C)** 6 d. (a) NC group. (b) MC group. (c) CMFP-H group. (d) CMFP-I group. (e) CMFP-F group. (f) PC group.

Compared with the NC group, the intestinal structure from the MC group was seriously damaged with the severely atrophic and conical intestinal villi, reduced goblet cells, and the unclear boundaries, the irregular distribution of epithelial cells, and severe eosinophilic infiltration, which lasted from 5 h to 6 d after injection (Figure 3A, Supplementary Figure 2A).

After 3 d of treatment (Figure 3B, Supplementary Figure 2B), compared with the MC group, the intestinal condition of CMFPs and PC groups were significantly improved, the intestinal structure was restored, the rupture of villi was improved, and the distribution of epithelial goblet cells was relatively regular. The therapeutic effect of CMFP-F was the most obvious. After 6 d of treatment (Figure 3C, Supplementary Figure 2C), the intestinal structure of the CMFPs and PC groups were recovered, with regular villi distribution and clear boundaries.

### Effects of CMFPs on oxidative stress in the intestine in colitis zebrafish

**Figure 4:**
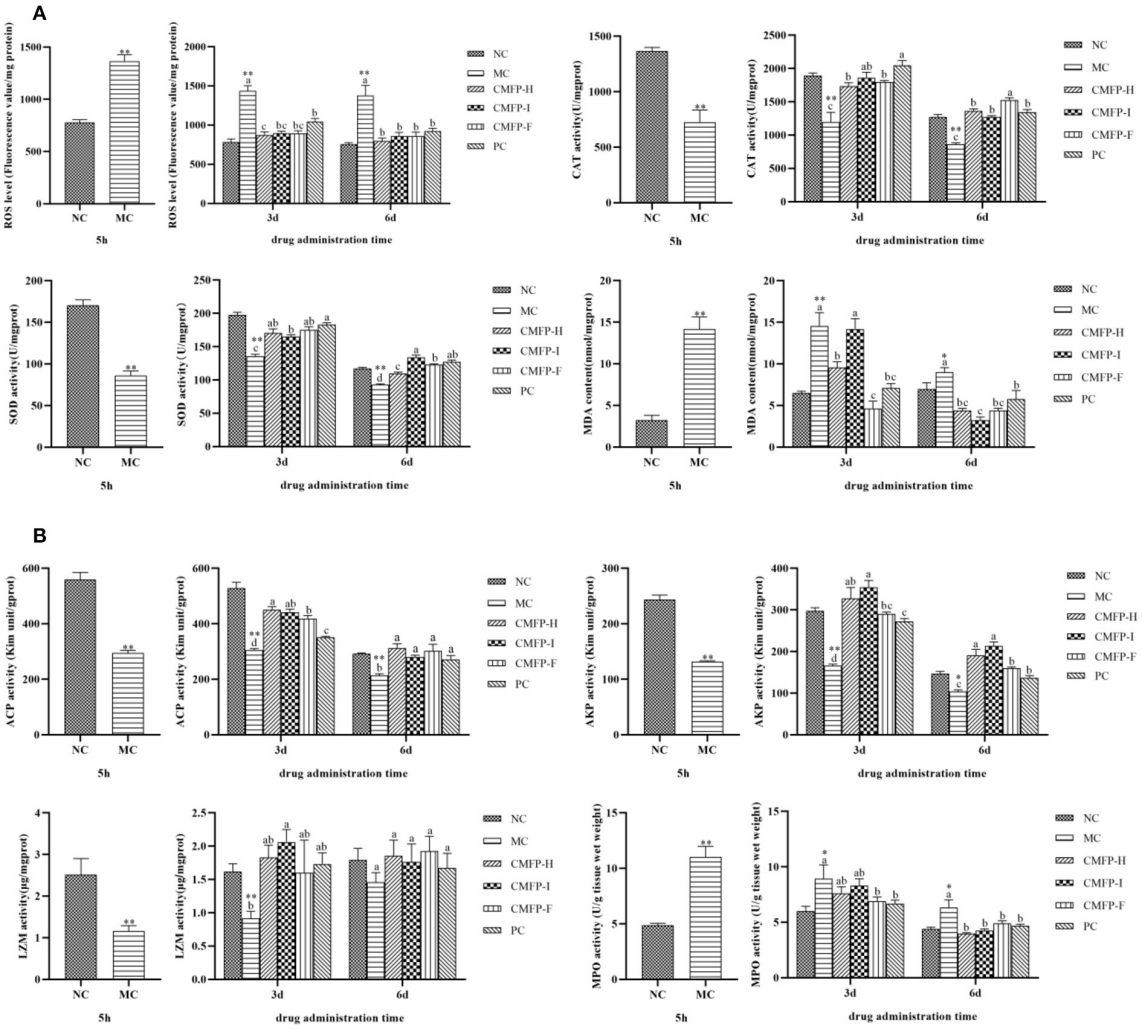
Effects of CMFPs on the activities of antioxidant enzymes and immune-related enzymes in the intestine of zebrafish with inflammatory bowel disease at 5 h, 3 d, and 6 d. **(A)** The ROS levels, SOD and CAT activities, and MDA content. **(B)** The ACP, AKP, LZM, and MPO activities. MC compared with NC, **p < 0.01, *p < 0.05; CMFPs and PC groups compared with MC indicated significant differences between groups marked with different letters (*p* < 0.05).

After rectal injection of oxazolone for 5 h in zebrafish, compared with the NC group, the levels of ROS and MDA in the MC group were increased by 75.40 and 339.26%, while the SOD and CAT activities were decreased by 49.56 and 46.73% (*p* < 0.01) (Figure 4A). This result indicated that the zebrafish colitis model was successfully established, which was consistent with Elmaksoud et al. (22).

After 3 d of treatment (Figure 4A), compared with the NC group, the ROS and MDA levels in the MC group were significantly increased by 83.70 and 128.68%, but the SOD and CAT activities were decreased by 31.50, 36.78% (*p* < 0.01). Compared with the MC group, the ROS and MDA contents in the three CMFPs groups and the PC group were decreased, while the activities of SOD and CAT were increased. Among them, the ROS content in the CMFP-H group was significantly reduced (39.20%, *p* < 0.05), and the CAT activity in the CMFP-I group was significantly increased (55.68%, *p* < 0.05). Meanwhile, the SOD activity in the CMFP-F group was significantly increased by 29.16%, and the MDA content was significantly decreased by 68.15% (*p* < 0.05).

After 6 d of treatment, compared with the NC group, the contents of ROS and MDA in the MC group were increased, while the activities of SOD and CAT were decreased. However, the SOD, CAT activities, and ROS contents in the CMFPs and PC treatment groups gradually approached normal levels, and the MDA contents were significantly lower than those in the NC group (Figure 4A). At this time, compared with the MC group, the SOD activity in the CMFP-I group was significantly increased (44.32%, *p* < 0.05), while the MDA and ROS contents were significantly decreased (64.19% and 37.63%, *p* < 0.05).

### Effects of CMFPs on the activities of immune-related enzymes

The effects of CMFPs on the activities of immune-related enzymes were further investigated in zebrafish (Figure 4B). After rectal injection of oxazolone for 5 h in zebrafish, compared with the NC group, the activities of ACP, AKP, and LZM of the intestinal tissues in the MC group were, respectively decreased by 47.28, 45.90, and 54.01% (*p* < 0.01). In contrast, the MPO activity was increased by 126.57% (*p* < 0.01).

After 3 d of treatment, compared with the NC group, the activities of ACP, AKP, and LZM in the MC group were decreased by 42.31, 44.07, and 43.45%, respectively (*p* < 0.01), while the MPO activity was increased by 48.95% (*p* < 0.05) compared with the NC group (Figure 4B). Compared with the MC group, the ACP, AKP, and LZM actions were increased, while the MPO activity was decreased in three CMFPs groups and the PC group. Among them, the ACP activity was increased significantly by 47.69% (*p* < 0.05) in the CMFP-H group. The AKP and LZM activities were increased significantly (112.99% and 125.05%, *p* < 0.05) in the CMFP-I group. The MPO activity was significantly decreased (22.77%, *p* < 0.05) in the CMFP-F group.

After 6 d of treatment (Figure 4B), compared with the MC group, the ACP activity in the CMFP-H group was increased (46.03%, *p* < 0.05) and the MPO level was significantly decreased (37.12%, *p* < 0.05), while the AKP activity in the CMFP-I group was markedly increased by 105.16% (*p* < 0.05) and the activity of LZM in the CMFP-F group was increased by 31.98%.

### Effects of CMFPs on the expression of inflammatory cytokines

**Figure 5:**
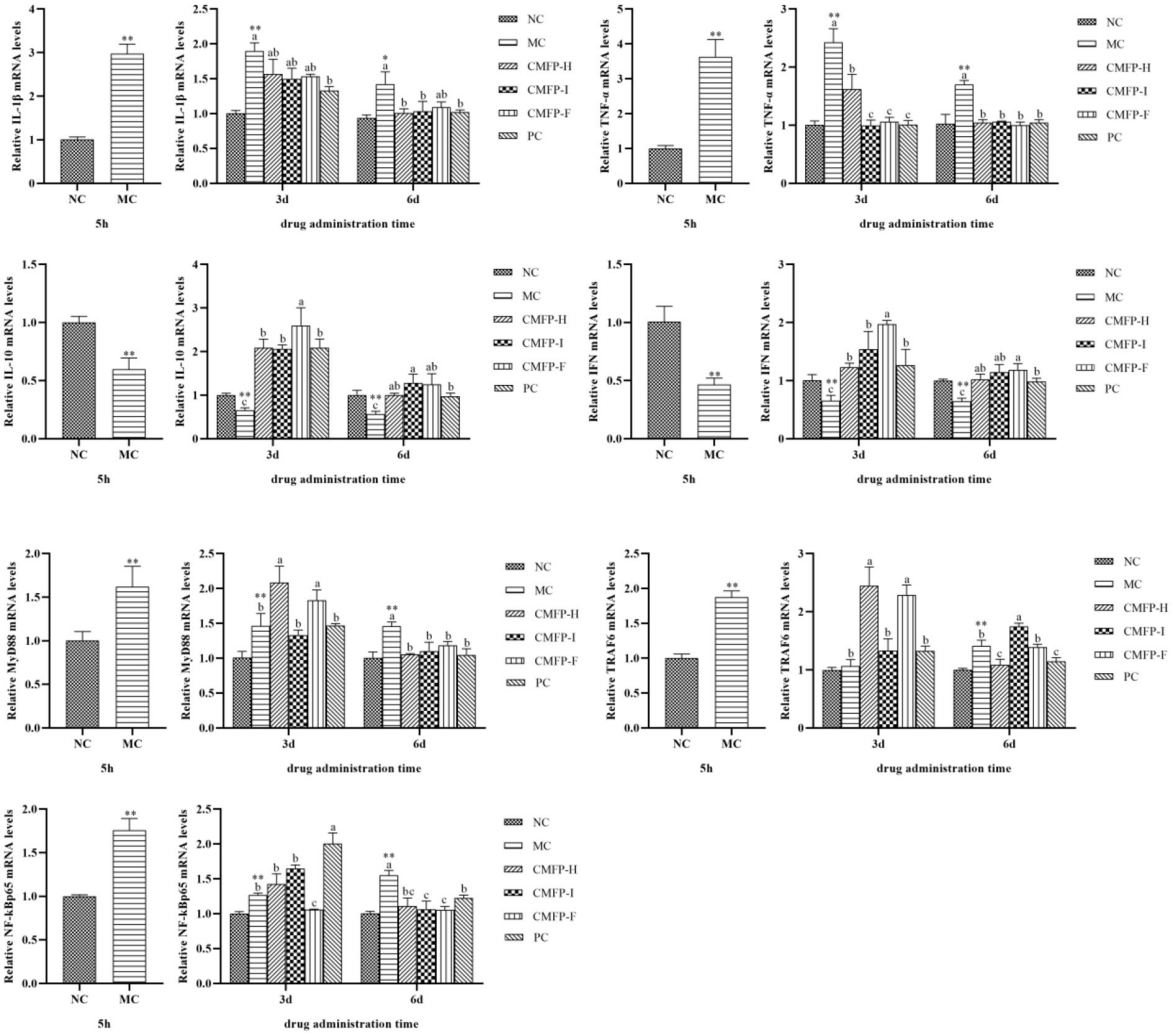
Effect of CMFPs on the expressions of inflammatory cytokines and MyD88/NF-κB Signaling Pathways in the intestine of zebrafish with inflammatory bowel disease at 5 h, 3 d, and 6 d. MC compared with NC, **p < 0.01, *p < 0.05; CMFPs and PC groups compared with MC indicated significant differences between groups marked with different letters (*p* < 0.05).

Compared with the NC group, the mRNA expression levels of *IL-1*β, *TNF-*α, *MyD88, TRAF6*, and NF-κB *p65* of the zebrafish intestinal tissue were significantly increased (*P* < 0.01), while the levels of *IL-10* and *IFN* were down-regulated (*p* < 0.01) (Figure 5) in the MC groups after 5 h of oxazolone injection. Compared with the MC group, the mRNA expression levers of *IL-1*β, *TNF-*α, *MyD88*, and NF-κB *p65* were significantly decreased (*p* < 0.05), while the expression levels of *IL-10* and *IFN* were relatively increased (*p* < 0.05) in the CMFPs group with CMFPs treatments at 6 d. At the same time, except that the expression of *TRAF6* in CMFP-I was significantly up-regulated, the mRNA expression levels of other genes were down-regulated in the CMFP-H and CMFP-F compared with the MC group (*p* < 0.05).

As shown in Figure 5, compared with the MC group, CMFPs treatment for 3 d could reduce the overexpression of *IL-1*β and *TNF-*α (*p* < 0.05), while the *IL-10* and *IFN* expression levels were all increased to varying degrees (*p* < 0.05). Among them, CMFP-I could significantly reduce the expression of pro-inflammatory cytokines (*IL-1*β and *TNF-*α), and CMFP-F could increase substantially the mRNA expression of anti-inflammatory cytokines (*IL-10* and *IFN*). However, there were differences in the face of three targets in the MyD88/NF-κB signaling pathway. Compared with the MC group, the mRNA levels of *MyD88, TRAF6*, and *p65* in the CMFP-H group were increased, the expression levels of *TRAF6* and *p65* in the CMFP-I group were up-regulated, while the expression of *MyD88* was down-regulated in the CMFP-I group (Figure 5). Consisted with this, the further result showed that the expression levels of *MyD88* and *TRAF6* were significantly increased (*p* < 0.05), but the expression of *p65* was significantly down-regulated (*p* < 0.05) in the CMFP-F group.

## Discussion

### Physicochemical properties of CMFPs

Rapid drying treatment is the last step in *C. militaris* industrial processes. It is also the initial processing link as a food raw material, which plays a vital role in controlling the quality of *C. militaris* fruiting body products. Meanwhile, accumulating evidence has shown that drying methods have a significant influence on the physicochemical properties and composition of materials, which in turn affects the biological activity of polysaccharides (8, 23). In this study, three polysaccharides, CMFP-H, CMFP-I, and CMFP-F, were extracted from *C. militaris* fruiting bodies under different drying treatments by hot water extraction method, respectively. Then, the extraction rate and main composition of CMFPs were further compared and analyzed. The data showed that the CMFP-I group had the highest extraction rate, followed by CMFP-F and CMFP-H groups. The result was somewhat different from that found in the polysaccharides from *Agaricus blazei* fruiting bodies previously reported, in which the polysaccharide yield of the freeze-drying method was significantly higher than that of vacuum drying and air drying (23). This difference might be related to the apparent difference in textures of fruiting bodies between Entomogenous and Agarics fungi.

Several previous studies have suggested that the polysaccharides were usually combined with other components such as uronic acids, phenolics, and proteins, thereby exhibiting various biological activities (23, 24). The data of this study demonstrated that the contents of neutral sugar, uronic acids, and total phenolic of CMFP-F were higher than that of CMFP-H and CMFP-I. However, the protein content of CMFP-F was significantly lower than that of CMFP-H and CMFP-I. This difference might be caused by the relatively high temperature, which might bring about organic carbohydrates and polyphenolic functional group destruction, in turn led to a decreasing in neutral sugar and uronic acid contents of extracted polysaccharides during HD and ID processes (24). And yet, the FD method can effectively avoid the loss of such heat-sensitive components in low temperature and vacuum (at −80°C and below 1 kPa). The surface ultrastructure characteristics of CMFPs also showed the CMFP-H had more uneven surface particle sizes with a small amount of debris and regular pore structures than CMFP-I and CMFP-F, which was similar to that of the polysaccharides from potato (25).

The data of our additional experiments showed that CMFP-H, CMFP-I, and CMFP-F were all composed of glucose, mannose, galactose, and glucosamine hydrochloride, each with different molar ratios. The result was different from the data of Jiang et al. (26), which showed that *C. militaris* polysaccharide was composed of glucose, galactose and mannose at molar ratios of 3.90:1.51:1. The differences in the monosaccharide composition of *C. militaris* polysaccharides may be due to differences in drying methods, material sources and extraction methods. ID treatment decreased glucose content while increasing mannose and galactose content in CMFPs compared with FD and HD. The reason may be that in the far-infrared radiation drying, the molecules in the material vibrate strongly, which accelerates the oxidation of hydroxyl groups and the break of intermolecular hydrogen bonds, and further affects the monosaccharide composition (9). To confirm these results, the changes in molecular weights (MW) and component separations of CMFP-H, CMFP-I, and CMFP-F were further detected. Our results showed that the peak times of the three CMFPs components were slightly different, but the peak positions were relatively consistent. However, there were also pronounced differences in MW and proportions of CMFP-F, CMFP-H, and CMFP-I. Among them, the relatively higher MW of CMFP-H might be due to the removal of the hydration layer of polysaccharides during the HD process, which could damage the structural integrity of polysaccharides, and in turn lead to polysaccharide aggregation (27, 28).

To verify the molecular weight results, the 0M and 0.1M fractions of three CMFPs were further separated using a Sephacryl S-400 Sephadex column in the present study. Our results demonstrated that the time and shapes of the peaks were similar among CMFP-H, CMFP-I, and CMFP-F components. Still, there significant differences in the peak size and the recoveries of each elements. Considering together, these above-mentioned findings suggested drying process had a great influence on the physicochemical properties of CMFPs and might produce important effects on their biological activities.

### Effects of CMFPs on oxidative stress in the intestine in colitis zebrafish

It is common knowledge that oxidative stress is one of the main factors involved in the development of the disease and its complications, as well as one of the main mechanisms involved in the IBD pathology (22). A large amount of evidence suggests that enteritis is associated with the imbalance between ROS and antioxidant activity. Overproduction of ROS or reduced antioxidant activity results in oxidative stress (29). At the same time, excess production of ROS can affect lipids, proteins, and nucleic acids, resulting in the formation of lipid peroxides, tissue damage, and an inflammatory cascade (30). In this study, we established a model of oxazolone-induced colitis in zebrafish. The data indicated that the intestinal structure characteristic of the oxazolone-induced zebrafish colitis was very similar to those of ulcerative colitis in humans (17). Our further results showed that CMFPs could significantly repair intestinal epithelial cell barrier damage, reducing neutrophil infiltration of the intestinal mucosa and further improving the morphology of intestinal tissue in zebrafish. Furthermore, CMFPs exhibited good antioxidant capacities, which could inhibit the generation of ROS and MDA, and increase the activities of antioxidant enzymes such as SOD and CAT to varying degrees. The result was similar to that of the compound *Cordyceps militaris* (CCM), which could improve dextran sulfate sodium (DSS) combined with high-fat-induced ulcerative colitis in mice (31).

In the present study, we also found MW detection peaks of CMFP-I components were the lowest. Recently, small molecular weight polysaccharides were reported to exert better antioxidant capacity than large molecular weight polysaccharides (32). Interestingly, our present results revealed that the SOD activity in the CMFP-I group was significantly increased compared with the MC group and associated with the most considerable reduction for the MDA and ROS contents at 6 d in the CMFP-I group. Considering recently reported studies together with our findings, these results demonstrated that different drying methods could have an essential influence on antioxidant activities of CMFPs in zebrafish with oxazolone-induced colitis.

### Effects of CMFPs on the activities of immune-related enzymes

Innate immunity is the most critical defense line of organisms against foreign invasion, and inflammatory response is the most important natural immune mechanism, during which inflammatory cells rapidly release a variety of proteolytic enzymes, including MPO and LZM, to regulate the occurrence of inflammation (33). Among them, LZM is ubiquitous in organisms and is an essential immune factor, which is usually used to determine the non-specific immune function of organisms (34). Meanwhile, excess ROS can improve the permeability of the lysosomal membrane and promote the release of its proteolytic enzymes, such as ACP and AKP, which are involved in the immune response of the body (35). As expected, our results revealed the activities of ACP, AKP, and LZM of the intestinal tissues in the MC group were, respectively decreased, while the MPO activity was increased in zebrafish with a rectal injection of oxazolone for 5 h compared with the NC group. The other results of this study indicated that CMFPs significantly reduced the level of MPO and enhanced the activities of ACP, AKP, and LZM in zebrafish. However, there were obviously different function effects on specific immune-related enzymes among CMFP-F, CMFP-H, and CMFP-I treatment groups.

### Effects of CMFPs on the expression of inflammatory cytokines

Severe tissue damage caused by inflammatory response is considered a key feature of colitis (36). In this study, our results showed that compared with the MC group, the mRNA expression levers of IL-1β and TNF-α were significantly decreased, while the expression levels of IL-10 and IFN were relatively increased in the three CMFPs groups at 6 d. In addition, this result was accompanied by a significant increment in the expression levels of MyD88, TRAF6, and NF-κB p65 in zebrafish treated with the three CMFPs for 6 d. Accumulating evidence suggests that IL-1β and TNF-α are typical pro-inflammatory cytokines that are rapidly increased when tissue injury or infection. IL-10 inhibits the production of chemokines, pro-inflammatory cytokines, and granulocyte survival mediators. It plays a crucial role in limiting the excessive inflammatory response caused by pathogenic infection to prevent host damage (37). The activation of NF-κB, which can mediate the expression of inflammatory mediators such as IL-1β, TNF-α, and IL-10, has been proved that can induce inflammatory factors that can further maintain and activate NF-κB, thereby aggravating inflammatory damage (38). MyD88 can participate in the regulation of IRAK and TRAF and phosphorylates IRAK to bind to TRAF6. The activated TRAF6 binds to MAP3K family member TAK-1 to activate TAK-1, which ultimately leads to the activation of NF-κB (39, 40). Overall, these results demonstrated that CMFPs could have better therapeutic effects on zebrafish colitis *via* activating the MyD88/NF-κB inflammatory signaling pathway, and thereby regulating inflammatory cytokines. Interestingly, our present results also showed that CMFP-H, CMFP-F, and CMFP-I exerted noticeable different regulation effects on the expression levels of IL-1β, TNF-α, IL-10, IFN, MyD88, and NF-κB p65, and this finding is quite rarely mentioned in the literature until now.

## Conclusion

In conclusion, there were significant differences in physicochemical properties of polysaccharide from *C. militaris* fruiting bodies under different drying methods. Further detailed histopathological and molecular detection suggested that CMFPs exhibited good antioxidant capacities, and could better improve oxazolone-induced zebrafish colitis *via* activating the MyD88/NF-κB inflammatory signaling pathway. However, we also found that there are obviously different function effects on specific immune-related enzymes and inflammatory cytokines among CMFP-F, CMFP-H, and CMFP-I treatment groups. The data might be helpful to future studies on effective and rapid drying methods for these macro-fungi and component separation for exploring CMFPs as functional food ingredients or complementary medicines of cytotoxic agents for the treatments of colitis.

## Data availability statement

The original contributions presented in the study are included in the article/Supplementary material, further inquiries can be directed to the corresponding authors.

## Ethics statement

The animal study was reviewed and approved by the Experimental Animal Ethics Committee of Shanxi Agricultural University.

## Author contributions

YW: investigation, validation, data curation, and writing-original draft. XD and YG: investigation and data curation. MC: resources and formal analysis. BD: software and visualization. JL: conceptualization, investigation, and writing-review and editing. JC: methodology, supervision, and writing-review. All authors contributed to the article and approved the submitted version.

## Funding

This study was financially supported by the National Natural Science Foundation of China General Fund Project (32072647) and the Key Scientific and Technological Innovation Team of Edible Fungi of Shanxi Province (201805D131009).

## Conflict of interest

The authors declare that the research was conducted in the absence of any commercial or financial relationships that could be construed as a potential conflict of interest.

## Publisher's note

All claims expressed in this article are solely those of the authors and do not necessarily represent those of their affiliated organizations, or those of the publisher, the editors and the reviewers. Any product that may be evaluated in this article, or claim that may be made by its manufacturer, is not guaranteed or endorsed by the publisher.
