# Supplementary material for: Elucidation of physicochemical properties of polysaccharides extracted from Cordyceps militaris fruiting bodies with different drying treatments and their effects on ulcerative colitis in zebrafish
Source: Front Nutr. 2022 Sep 2;9:980357. doi: 10.3389/fnut.2022.980357 (PMC9481070; doi:10.3389/fnut.2022.980357)
Supplement: Supplementary file 1 [file Data_Sheet_1.docx]

**SUPPLEMENTARY MATERIAL**

**SUPPLEMENTARY TABLE 1.** The gene primer sequences were used for RT-qPCR.

**SUPPLEMENTARY TABLE 2.** Comparative data of molecular weight determination of CMFPs.

**SUPPLEMENTARY TABLE 3.** The contents and recovery rate of each component in DEAE-52 of CMFPs.

**SUPPLEMENTARY TABLE 4.** The contents and recovery rate of each component in Sephacryl S-400 of CMFPs.

**SUPPLEMENTARY FIGURE 1.** Ion chromatogram of monosaccharide mixture and CMFPs. (M) monosaccharide mixture. (I) CMFP-I. (H) CMFP-H. (F) CMFP-F.

**SUPPLEMENTARY FIGURE 2.** Effect of CMFPs on the histopathological structure (AB-PAS staining) of intestinal in inflammatory bowel disease zebrafish. (A) 5 h. (B) 3 d. (C) 6 d. (a) NC group. (b) MC group. (c) CMFP-H group. (d) CMFP-I group. (e) CMFP-F group. (f) PC group.

**SUPPLEMENTARY TABLE 1.** The gene primer sequences were used for RT-qPCR.

| Primer name | Primer sequences of Forward(F) and Reverse(R) (5’**→**3’) | Product size(bp) |
| --- | --- | --- |
| β-actin | F:CCATTGAGCACGGTATTG  R:CTGTTGGCTTTGGGATTC | 139 |
| IL-1β | F:CCCAATCCACAGAGTTTG  R:GTAAGACGGCACTGAATC | 101 |
| TNF-α | F:TGGTGTCTAGGAGGAAAG  R:GTCTTATGGAGCGTGAAG | 115 |
| IFN | F:CTACTTGCGAATGGCTTG  R:TCCTCCACCTTTGACTTG | 145 |
| IL-10 | F:CATTTGTGGAGGGCTTTC  R:TGACGTGACATCCATAGG | 150 |
| NF-KB p65 | F:GGCTACTATGAGGCAGAT  R:CCACATCCTTCTTCTTCAC | 185 |
| TRAF6 | F:TCTCCGCTCGGCAGTACAGA  R:TCCTCAAGCAGCACCTCGTT | 117 |
| MyD88 | F:GTGTGGACCATCGCCAGTGA  R:TCCTGGGCAAAGACTGAGCG | 129 |

**SUPPLEMENTARY TABLE 2.** Comparative data of molecular weight determination of CMFPs.

| Samples | Peak 1 | | | | | |
| --- | --- | --- | --- | --- | --- | --- |
|  | RT(min) | Mp | Mw | Mn | Mw/Mn | Percentage ratio(%) |
| CMFP-I | 33.136 | 7.27453×10^5^ | 10.99848×10^5^ | 5.71883×10^5^ | 1.9232 | 50.118 |
| CMFP-H | 32.59 | 9.21641×10^5^ | 14.19080×10^5^ | 7.19189×10^5^ | 1.9732 | 63.796 |
| CMFP-F | 32.727 | 8.68518×10^5^ | 13.31181×10^5^ | 6.78997×10^5^ | 1.9605 | 73.166 |
| Samples | Peak 2 | | | | | |
|  | RT(min) | Mp | Mw | Mn | Mw/Mn | Percentage ratio(%) |
| CMFP-I | 41.116 | 2.2907×10^4^ | 2.6533×10^4^ | 2.0070×10^4^ | 1.3220 | 49.882 |
| CMFP-H | 41.037 | 2.3705×10^4^ | 2.7530×10^4^ | 2.0747×10^4^ | 1.3269 | 36.204 |
| CMFP-F | 40.993 | 2.4161×10^4^ | 2.8101×10^4^ | 2.1134×10^4^ | 1.3297 | 26.834 |

**SUPPLEMENTARY TABLE 3.** The contents and recovery rate of each component in DEAE-52 of CMFPs.

| Samples | CMFP-H | CMFP-I | CMFP-F |
| --- | --- | --- | --- |
| CMFP mass (mg) | 958.7 | 650.7 | 930.1 |
| 0M mass (mg) | 497.8 | 353.5 | 479.2 |
| 0.1M mass (mg) | 151.9 | 123.4 | 207.0 |
| Total mass (mg) | 649.7 | 476.9 | 686.2 |
| The recovery rate of 0 M | 0.7662 | 0.7412 | 0.6983 |
| The recovery rate of 0.1 M | 0.2338 | 0.2588 | 0.3017 |
| The total recovery rate | 0.6777 | 0.7329 | 0.7378 |

**SUPPLEMENTARY TABLE 4.** The contents and recovery rate of each component in Sephacryl S-400 of CMFPs.

| Samples | CMFP-H_0_ | CMFP-I_0_ | CMFP-F_0_ | CMFP-H_1_ | CMFP-I_1_ | CMFP-F_1_ |
| --- | --- | --- | --- | --- | --- | --- |
| CMFP mass (mg) | 102.2 | 100.1 | 102.0 | 51.1 | 49.8 | 50.0 |
| α mass (mg) | 28.2 | 19.7 | 17.0 | 16.0 | 16.8 | 23.2 |
| β mass (mg) | 25.1 | 40.1 | 33.6 | 6.7 | 6.2 | 1.2 |
| Total mass (mg) | 53.3 | 59.8 | 50.6 | 22.7 | 23 | 24.4 |
| The recovery rate of α | 0.5291 | 0.3294 | 0.3360 | 0.7048 | 0.7304 | 0.9508 |
| The recovery rate of β | 0.4709 | 0.6706 | 0.6640 | 0.2952 | 0.2696 | 0.0492 |
| The total recovery rate | 0.5215 | 0.5974 | 0.4961 | 0.4442 | 0.4618 | 0.4880 |

**F**


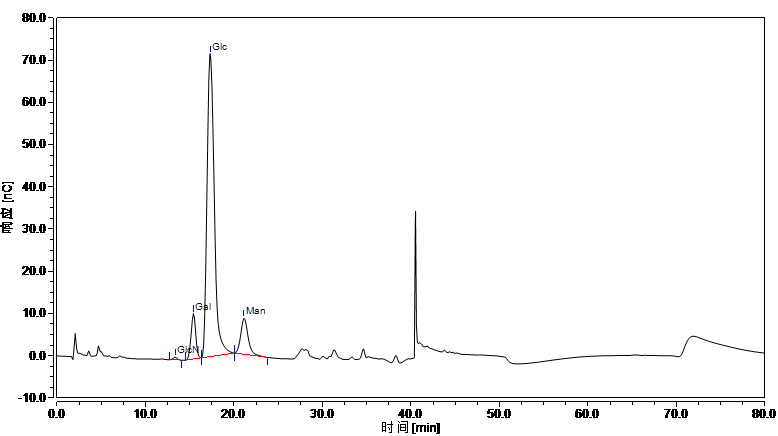


Time (min)

The peak height (nc)

**H**


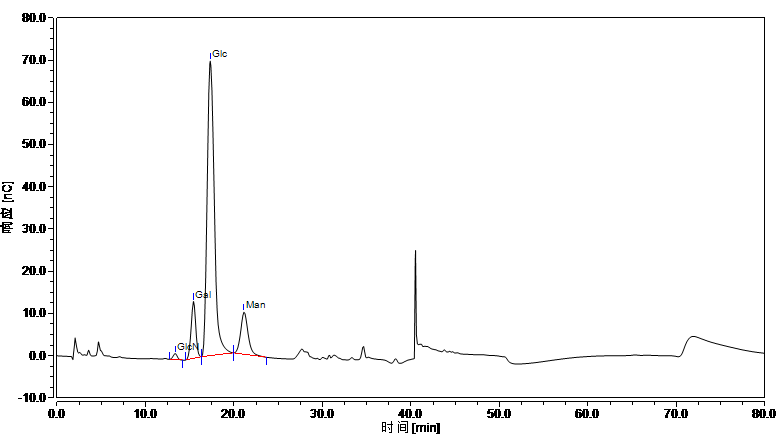


Time (min)

The peak height (nc)

**I**


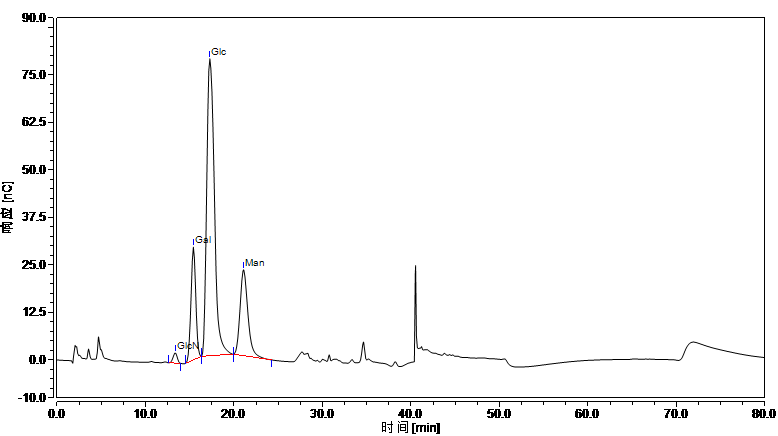


Time (min)

The peak height (nc)


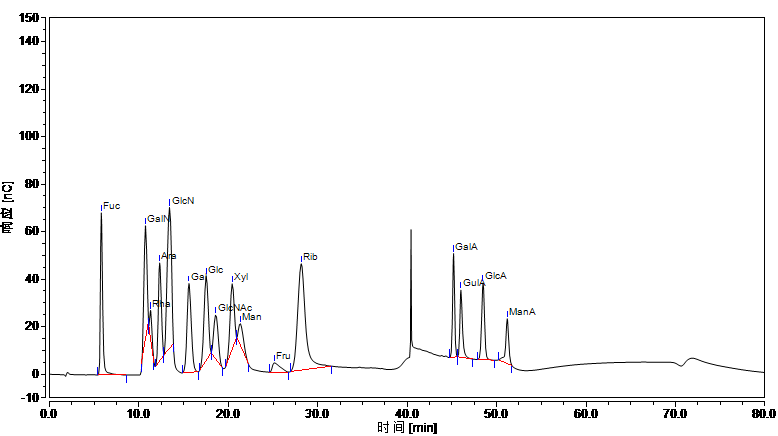


Time (min)

The peak height (nc)

**M**

**SUPPLEMENTARY FIGURE 1.** Ion chromatogram of monosaccharide mixture and CMFPs. (M) monosaccharide mixture. (I) CMFP-I. (H) CMFP-H. (F) CMFP-F.


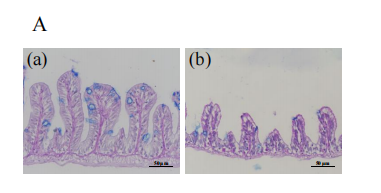

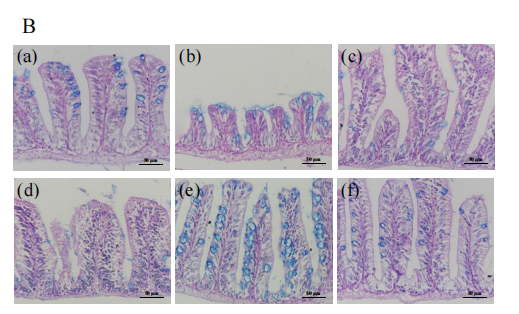

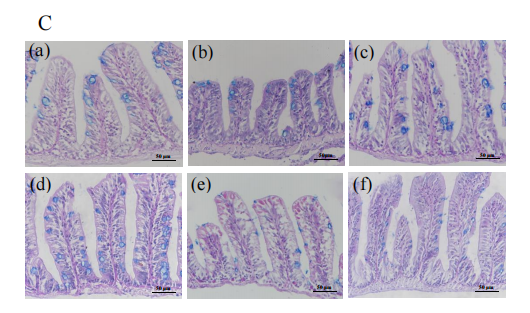


**SUPPLEMENTARY FIGURE 2.** Effect of CMFPs on the histopathological structure (AB-PAS staining) of intestinal in inflammatory bowel disease zebrafish. (A) 5 h. (B) 3 d. (C) 6 d. (a) NC group. (b) MC group. (c) CMFP-H group. (d) CMFP-I group. (e) CMFP-F group. (f) PC group.
